# A Call to Action: Hypertensive Crises, Non-ST-Elevation Myocardial Infarction (NSTEMI), and Heart Failure in the Early Twenties

**DOI:** 10.7759/cureus.40156

**Published:** 2023-06-08

**Authors:** Stefan Milutinovic, Slobodan Lazarevic, Miljan Krstovic, Abraham Bell

**Affiliations:** 1 Internal Medicine, Florida State University College of Medicine, Cape Coral, USA; 2 Internal Medicine, University of Niš Faculty of Medicine, Niš, SRB; 3 Digestive Surgery, University of Niš Faculty of Medicine, Niš, SRB

**Keywords:** inflammation and heart failure, hypertensive crisis, heart failure, non-st elevation myocardial infarction (nstemi), extreme obesity

## Abstract

A 21-year-old obese male with multiple hypertensive crises was diagnosed with non-ST-elevation myocardial infarction (NSTEMI), leading to heart failure due to uncontrolled hypertension and medication noncompliance. The patient's morbid obesity likely contributed to undiagnosed chronic hypertension, increasing the risk of atherosclerosis and cardiovascular diseases. Morbid obesity leads to increased interleukin-6 levels, promoting plaque accumulation and rupture. Obesity also triggers a pro-inflammatory and prothrombotic state, characterized by elevated levels of serum high-sensitivity C-reactive protein (hs-CRP), plasminogen activator inhibitor 1 (PAI-1), and other cytokines. This inflammatory state contributes to atherosclerosis development and renders plaques more prone to rupture. Additionally, obesity has been shown to increase the size of coronary thrombosis once the plaque ruptures. Treating obesity is crucial for the patient's well-being and reduces the burden on healthcare systems and society. Establishing a strong physician-patient relationship is essential for motivating lifestyle modifications, which are often the primary treatment approach for obesity and its complications.

## Introduction

A hypertensive crisis is a sudden increase in blood pressure, typically with systolic blood pressure (SBP) >180 mmHg or diastolic blood pressure (DBP) >120 mmHg. Medication noncompliance and obesity are risk factors for this condition [1]. Hypertensive emergency and hypertensive urgency can result in severe complications including stroke, myocardial infarction, and heart failure, even in young adults [2]. 

## Case presentation

A 21-year-old obese (BMI of 49.5kg/m^2^) and active male smoker without a significant medical or family history of premature coronary artery disease (CAD) presented to the emergency department with orbital cellulitis and high blood pressure (225/135). The initial electrocardiogram revealed signs of left ventricular hypertrophy (LVH) and troponin levels were elevated (0.041ng/ml) due to demand ischemia. Echocardiography showed grade II diastolic dysfunction with ejection fraction (EF) >60%, and thyroid stimulating hormone (TSH) and urinalysis were within normal limits. The patient was admitted for his chief complaint and successfully managed for hypertensive emergency. The patient was subsequently discharged on lisinopril and hydralazine with a scheduled outpatient visit.

**Figure 1:**
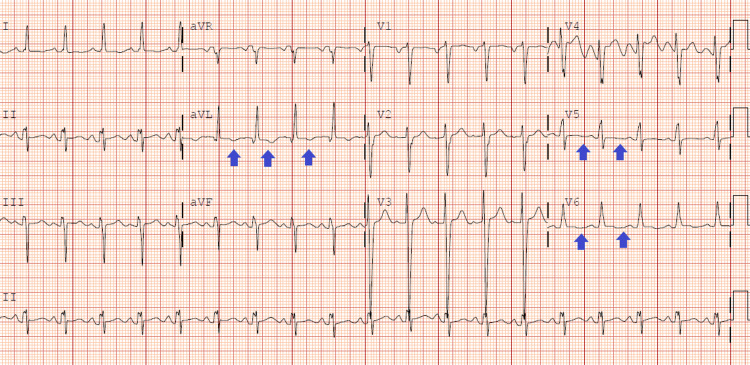
Electrocardiography showing T wave abnormalities in lateral leads (blue arrows)

Over the next three years, the patient had multiple uncontrolled blood pressure visits due to medication noncompliance and was admitted numerous times to the hospital with a chief complaint of chest pain. His BMI throughout this time remained in the range of morbid obesity. On a subsequent encounter, he presented to the emergency department with chest pain, dyspnea, nausea, vomiting, and visual changes. The patient denied any substance abuse. His lipid profile was normal with mild elevation in low-density lipoprotein (LDL) at 109 mg/dl. His glycated hemoglobin (HbA1c) was in the prediabetes range at 5.8%. The hypercoagulable profile was unremarkable. The initial electrocardiogram showed T wave abnormalities in lateral leads (Figure 1), and troponin levels were elevated at 17 ng/ml and 23 ng/ml, respectively.

**Figure 2:**
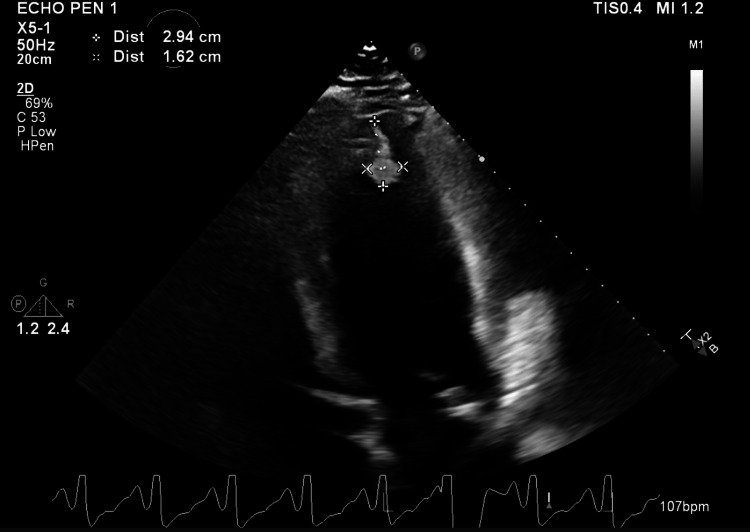
Echocardiography showing presence of apical mural thrombus

**Figure 3:**
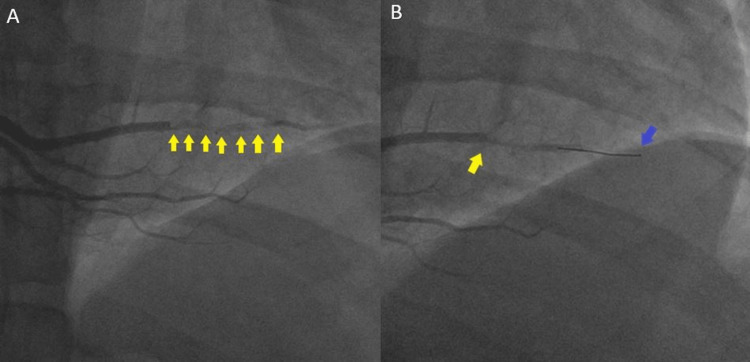
Cardiac catheterization. (A) PDA clot (yellow arrows); (B) PDA clot (yellow arrow) and coronary guidewire (blue arrow) PDA: posterior descending artery

The patient was diagnosed with non-ST segment elevation myocardial infarction (NSTEMI) and echocardiography findings showed combined systolic and diastolic dysfunction (EF=34%) with an apical mural thrombus (Figure 2). Cardiac catheterization revealed an extensive clot in the posterior descending artery (PDA) and a thrombectomy was then performed (Figure 3). The patient was started on dual antiplatelet therapy and apixaban. Screenings for secondary causes of hypertension including the aldosterone/renin ratio and urine metanephrines levels were unremarkable.

The patient was discharged in stable condition, on multiple anti-hypertensive medications, and has well-controlled blood pressure. At present, he reports compliance with his guideline-directed medical therapy and has been regularly seen by his cardiologist. His EF recovered in three months to >55%. 

## Discussion

Our case highlights the devastating complications that uncontrolled hypertension can cause in young adults. This man in his early twenties had undiagnosed chronic hypertension, which may have resulted from his morbid obesity. Research shows that young adults are more likely to have undiagnosed hypertension, even with regular primary care visits [3]. Unfortunately, his disease progressed and he experienced hypertensive crises, NSTEMI, and mixed systolic-diastolic heart failure due to medication noncompliance. Even in younger patients, increased interleukin-6 caused by morbid obesity has been linked to plaque accumulation and rupture [4]. His obesity and hypertensive shearing forces may explain the PDA clot. This case underscores the need for prevention and close monitoring of obesity, as well as patient education on medication adherence.

Obesity is an important risk factor for atherosclerosis. There is a 10% increase in the risk for atherosclerosis and coronary heart disease for each one-point increase in BMI above normal weight [5]. In obesity, there is a decrease in anti-inflammatory adipokines (adiponectin and omentum-1) and an increase in proinflammatory adipokines (retinol-binding protein 4, leptin, interleukin-6, monocyte chemoattractant protein-1, resistin), free fatty acids, and inflammatory cells. These factors lead to vascular inflammation and cell dysfunction, which contribute to the progression of atherosclerosis [6-8].

Increases in pro-inflammatory adipokines and fatty acids, resistance to insulin, and impairment in baroreceptor function trigger an increase in sympathetic nervous activation. Activation of the sympathetic nervous system and the renin-angiotensin-aldosterone system is also promoted by increased leptin and leptin receptor signaling which is found in obesity [9,10]. Leptin also causes increased secretion of neprilysin from adipocytes and the kidneys, which causes a deficiency of endogenous natriuretic peptides in obese individuals with resultant additional sodium retention and plasma volume expansion. Increased concentrations of aldosterone, neprilysin, and leptin together with increased sympathetic nerve activity lead to hypertension, plasma volume expansion, sodium retention, vascular endothelial dysfunction, and ultimately atherosclerosis [11].

Obesity is associated with a pro-inflammatory and prothrombotic state, characterized by elevated levels of serum high-sensitivity C-reactive protein (hs-CRP), plasminogen activator inhibitor 1 (PAI-1), and numerous other cytokines [12-17]. This pro-inflammatory state contributes to the development of atherosclerosis and makes plaques more susceptible to rupture [18,19]. Furthermore, it is proposed that the prothrombotic state associated with obesity may increase the size of coronary thrombosis once the plaque has ruptured. As a result, obesity increases the risk of major adverse cardiovascular events (MACE) beyond what is expected based on traditional risk factors [20].

Evidently, obesity poses an important risk factor for the development of a wide array of diseases, especially cardiovascular. Thus, treating obesity is crucial not only for the patient's well-being but also to reduce the socioeconomic burden on the healthcare system and society. A strong physician-patient relationship is crucial for establishing and maintaining a patient's motivation for lifestyle modifications, which are often the first line of treatment for obesity and its complications. Reducing sugar and saturated fatty acid intake can lead to a decrease in inflammation and endothelial cell dysfunction mediated by increased activation of pro-inflammatory cytokines. Consuming a Mediterranean diet, which includes increased consumption of fruits, whole grains, and vegetables, improves the quality of life for obese patients. In addition, it reduces triglyceride and total cholesterol levels and increases high-density lipoprotein (HDL) levels, thereby decreasing the risk for adverse cardiovascular events and often eliminating the need for lipid-lowering therapy [21].

Physical exercise is an important aspect of a successful obesity treatment plan in addition to dietary modifications. Moderate-intensity aerobic exercise, recommended for at least 150 minutes weekly, can yield satisfactory results for patients. Strength/resistance training can also improve weight loss and physical fitness by increasing lean body mass [21-23]. Moreover, exercise regimens combined with dietary changes can help improve other conditions such as diabetes and hypertension, which often coexist with obesity [24,25]. A thorough review of the patient’s medication list may contribute to favorable results since many drugs are associated with an increase in weight and may have appropriate alternatives that are not associated with such effects [26].

Pharmacological treatment can be considered for patients who don't achieve satisfactory results with lifestyle modifications alone. There are several classes of weight loss medications such as glucagon-like peptide (GLP) analogs, adrenergic drugs, and pancreatic lipase inhibitors, each with its unique adverse effect profile. The choice of medication should be based on the patient's preferences and the physician's consideration of the patient's distinctive characteristics.

In the event that the aforementioned treatment methods have proven to be ineffective, despite the person’s motivation for change, surgery may offer additional therapeutic modalities for such patients. Specifically, bariatric procedures such as a sleeve gastrectomy and gastric bypass surgery have proven to be efficacious in lowering visceral fat mass by around 40%, as well as improving insulin sensitivity and glucose metabolism, along with reductions in HbA1c levels in patients with type 2 diabetes [27-29]. These operations are associated with a relatively low risk of complications, the most common being anastomotic leak and fistula formation with the associated peritonitis, as well as more rare complications such as deep venous thromboses and thromboses in the portal vein system. As far as metabolic and nutritional complications are concerned, acid-base and electrolyte disturbances, along with certain nutritional deficiencies can be expected, with the most severe being hypoglycemia and osteoporosis [30-32].

## Conclusions

Hypertensive crises and premature onset of the first NSTEMI are possible in young adults without known secondary causes. Excessive obesity is a significant risk factor for these conditions, highlighting the importance of prioritizing prevention and close monitoring of obesity in young patients. Additionally, medication compliance is crucial in managing hypertension and reducing the risk of cardiovascular events. Healthcare professionals should reinforce the importance of medication adherence to young patients to ensure optimal control of hypertension and minimize the risk of hypertensive crises and cardiovascular complications. Early detection and management of hypertension, along with lifestyle modifications and medication adherence, are critical for preventing these conditions and improving long-term cardiovascular outcomes in young adults.
